# Les infections à *Pseudomonas aeruginosa* au service des maladies infectieuses du CHU YO, Burkina Faso: à propos deux cas

**DOI:** 10.11604/pamj.2015.21.78.4739

**Published:** 2015-05-29

**Authors:** Savadogo Mamoudou, Dao Lassina, Koueta Fla

**Affiliations:** 1 Service des Maladies infectieuses CHU YO, Ouagadougou, Burkina Faso; 2 CHU Pédiatrique Charles De Gaulle, Ouagadougou, Burkina Faso

**Keywords:** Pseudomonas aeruginosa, meningite, infection urinaire, Pseudomonas aeruginosa, meningitis, urinary tract infection

## Abstract

Nous rapportons deux cas d'infection à *Pseudomonas aeruginosa*: un cas de méningite et un cas d'infection urinaire. Les auteurs rappellent qu’à côté des étiologies classiques des méningites et des infections urinaires, des germes résistants comme *Pseudomonas aeruginosa* peuvent être responsables d'infections à localisation méningées et urinaires et dont il faut connaître pour une bonne prise en charge. Le traitement de ces infections requiert un antibiogramme au regard de la grande capacité de résistance de *Pseudomonas aeruginosa* en milieu hospitalier. La limitation des gestes invasifs et l'application rigoureuse des mesures de prévention des infections en milieu hospitalier contribueront à lutter efficacement contre ces infections en milieu de soins.

## Introduction

Malgré les progrès thérapeutiques, la mortalité due aux infections à *Pseudomonas aeruginosa* reste élevée à cause d'une part, des difficultés thérapeutiques engendrées par cette bactérie et d'autre part, à cause des pathologies associées [1]. Ce sont des infections opportunistes qui posent des problèmes préoccupant dans nos hôpitaux en raison de leur fréquence et de leur résistance aux antibiotiques [2]. Les infections à *Pseudomonas aeruginosa* sont des évènements cliniques rares et sévères, très souvent décrites dans un cadre nosocomiale [3–6]. A travers deux cas cliniques, les auteurs se fixent pour objectif de déterminer les caractéristiques cliniques et bactériologiques de ces infections.

## Patient et observation

### Patient 1

I. M. âgé de 9 ans, est suivi depuis sept ans au service d'ORL du centre hospitalier Yalgado Ouédraogo pour otite moyenne chronique purulente à *Pseudomonas aeruginosa* compliquée de perforation tympanique. Il a été admis au Centre hospitalier universitaire Yalgado Ouédraogo pour céphalées, agitations le 21/06/2013. L'examen notait, un état général altéré, une conscience obnubilée, un syndrome infectieux (T 38,9°, pouls = 158 cycles/mn), un syndrome méningé franc (raideur de la nuque, Kernig et Brudzinski présents) et une otite bilatérale avec perforation tympanique droite. Sur le plan biologique, l'examen du pu auriculaire avait isolé Candidas non albicans et *Pseudomonas aeruginosa* résistant à la ceftriaxone, à l'amoxicilline + acide clavulanique, à la pipéracilline et intermédiaire à la ticarcilline, mais sensible à la gentamicine, à l'amikacine, à la nétilmicine, à la ciprofloxacine et à la ceftazidime. L'examen du liquide céphalo rachidien (LCR) avait montré à la cytologie plus de 1000 leucocytes/mm^3^. L'examen bactériologique du LCR était revenu négatif, tandis qu’à l'examen biochimique on notait une hypoglycorachie (0,2 mmol/l) et une hyperproteinorachie (4,57g/l). L'hémogramme montrait une hyperleucocytose avec des globules blancs à 17 800/mm^3^. Sur le plan thérapeutique, la ceftriaxone 3g/j + la gentamicine 100 mg/j, en plus du traitement institué en ORL (Oflocet) avait été administré mais le 28/06/2013, il décédait dans un tableau d'agitation psychomotrice accompagnée de céphalées et d'une otorrhée muco purulente striée de sang à l'oreille droite.

### Patient 2

DA 42 ans éleveur de porc ayant un antécédent de poliomyélite, forme paralytique à l'enfance, a été admis le 16/05/2013 aux urgences médicales du Centre hospitalier universitaire Yalagado Ouédraogo, pour convulsions fébriles chez qui une ponction lombaire réalisée avait ramené un liquide céphalo rachidien clair avec à la cytologie 26 éléments nuclées/mm3. L'examen biochimique du liquide céphalo rachidien a noté une glucorachie et une proteinorachie normales. L'hémoculture, l'ECBU et la goutte épaisse réalisés également à l'entrée étaient revenus négatifs. La tomodensitométrie cérébrale avait objectivée un kyste arachnoïdien temporal antérieur droit évoquant une cysticercose et la sérologie VIH est revenue négative. Sous traitement, l’évolution a été marquée par l'apparition de crises tonico cloniques et d'une aphasie qui motivèrent son transfert au service de réanimation où il a séjourné pendant huit jours. Un examen cytobactériologique des urines (ECBU) demandé à son retour de la réanimation, devant une pyurie constatée au niveau de la sonde urinaire avait isolé *Pseudomonas aeruginosa* sensible à la ceftazidime, à l'imipénème, à la ticarcilline, à la tobramycine, mais résistant à l'ampicilline, à amoxicilline + acide clavulanique, à la ciprofloxacine, à la ceftriaxone, à l'acide nalidixique et au cotrimoxazole. La ceftazidime avait été prescrite en raison de 2g/ j et il a été libéré le 07 Juillet 2013 après 52 jours d'hospitalisation.

## Discussion

Les infections à *Pseudomonas aeruginosa* sont ubiquitaires, opportunistes très souvent décrites dans un cadre nosocomiale. *Pseudomonas aeruginosa* a un mécanisme de résistance qui est soit native, soit acquise [7, 8]. C'est le premier agent pathogène des otites externes [6, 9] et il occupe la deuxième et la cinquième place des bactéries responsables d'infections nosocomiales respectivement en Europe et Aux Etats-Unis [1]. Les méningites bactériennes représentent une importante cause de morbidité et de mortalité des enfants dans les pays en voie de développement [10]. Elles sont souvent d'origine otogène, par contigüité ou après une phase de bactériémie [3]. Le milieu hospitalier constitue une source majeure de contamination croisée par *Pseudomonas aeruginosa* [2] et il est important de renforcer les mesures de prévention des infections lors des gestes thérapeutiques en l'occurrence lors des poses de sondes urinaires [11]. L'infection urinaire à *Pseudomonas aeruginosa* a été diagnostiquée après un séjour au service de réanimation. La réanimation est en effet connue pour être la discipline médicale où les infections nosocomiales sont les plus fréquentes; c'est dit-on l’« épicentre de la résistance aux antibiotiques ». Cette situation est due entre autres à la conjonction de la fréquence d´utilisation des dispositifs invasifs [12], lors des gestes thérapeutiques en l'occurrence lors des poses de sondes urinaires [11]. Selon Leone, l´infection urinaire nosocomiale chez un patient ayant une sonde vésicale est le reflet d´une politique générale d´hygiène, allant des soins infirmiers lors de la pose de la sonde jusqu´à la gestion rigoureuse de l´écologie du service [13]. Candidas non albicans avait également été isolé dans le pus auriculaire du premier patient, corroborant ainsi les constats de Mear qui faisait remarquer que Pseudomona aeruginosa et Candidas étaient des pathogènes opportunistes, coexistant fréquemment, s'influençant mutuellement, et responsables d'infections chez les patients prédisposés [14]. *Pseudomonas aeruginosa* était résistante à la pipéracilline et intermédiaire à la ticarcilline, alors qu'elle est habituellement sensible aux uréidopénicillines et aux carboxypénicillines [4, 8, 15]. Ce qui témoigne d'une résistance acquise favorisée par la pression de sélection après plusieurs années de suivi en ORL. Les signes cliniques de la méningite à *Pseudomonas aeruginosa*, ne différaient pas des autres types de méningite, et l'analyse du LCR n'avait rien de spécifique. Cependant son traitement dure plus longtemps (20 jours) [16] et fait appel habituellement à la ceftazidime associée à l'amikacine [4]. Mais Brion préfère une association fosfomycine + ciprofloxacine [5]. L'association habituelle d'antibiotiques a pour objectif d’élargir le spectre d'action, d'accroître la vitesse de la bactéricidie et de limiter l’émergence de souches bactériennes résistantes [3, 16, 17]. *Pseudomonas aeruginosa* n´était sensible qu´à quelques antibiotiques et le traitement des infections par le bacille pyocyanique restent, à l´heure actuelle, un problème préoccupant. En effet, un traitement antibiotique même bien conduit, surtout s´il est tardif, n´entraîne pas toujours la guérison. C´est pourquoi l´effort dans la lutte contre le bacille pyocyanique doit passer en premier lieu par la prévention [8]. Le pronostic des méningites à *Pseudomonas aeruginosa* est sévère [3, 7, 8, 18] et les signes qui ont précédé le décès de notre cas de méningite, nous autorisent à émettre d'autres hypothèses diagnostiques comme un cholesteatome ou une otite maligne à l'origine de la méningite.

## Conclusion

Les infections à *Pseudomonas aeruginosa* sont redoutables à cause de leur capacité de résistance aux antibiotiques. Leur traitement nécessite un diagnostic précis et un choix rationnel d'antibiotiques basé sur un antibiogramme. Leur prévention passe par une prise en charge correcte des otites, la limitation des gestes invasives, l'application rigoureuse des mesures de prévention des infections dans les services.

## References

[1] Alaoui R, Benchekroun FL, Jazouli N (1994). Les abcès cérébraux d'origine otogène: à propos de 35 cas. Revue de laryngologie, d'otologie et de rhinologie..

[2] Bertrand X, Blasco G, Belle E (2003). Importance de la transmission croisée dans l’épidémiologie de Pseudomonas aeruginosa en service de soins intensifs. Annales Françaises d'Anesthésie et de Réanimation..

[3] Kienlen J (1998). Infections à pyocyaniques en réanimation. Conférences d'actualisation..

[4] Lambert PA (2002). Mechanisms of antibiotic resistance in Pseudomonas Aeruginosa. Journal of the royal society of medicine..

[5] Brion JP, Morand P, Petitjean O (2000). Traitement des méningites expérimentales à Pseudomonas aeruginosa avec la ciprofloxacine et la fosfomycine. Medecine et Maladies Infectieuses..

[6] Juhi T, Bibhabati M, Archana T (2009). Pseudomonas aeruginosa meningitis in post neurosurgical patients. Neurology Asia..

[7] Mesaros N, Nordmann P, Plésiat P (2007). Pseudomonas aeruginosa: résistance et options thérapeutiques à l'aube du deuxième millénaire. Antibiotiques..

[8] Bricha S, Ounine K, Oulkheir S (2009). Facteurs de virulence et epidemiologie lies nau pseudomonas aeruginosa. Revue Tunisienne d'Infectiologie..

[9] CMIT (2012). Infections à Pseudomonas. In E Pilly: Vivactis Plus Ed..

[10] Ophélie Marais (2011). Traitement par ceftriaxone des méningites bactériennes chez l'enfant. Actualités pharmaceutiques hospitalières..

[11] Boutiba-Ben Boubaker I, Boukadida J, Triki O (2003). Épidémie d'infections urinaires nosocomiales à Pseudomonas aeruginosa multirésistant aux antibiotiques. Pathologie Biologie..

[12] Dia NM, Ka R, Dieng C, Diagne R ML, Dia L, Fortes BM, Diop AI, Sow PS (2008). Résultats de l'enquête de prévalence des infections nosocomiales au CHNU de Fann (Dakar, Sénégal). Médecine et Maladies Infectieuses..

[13] Léone M, Arnaud S, Boisson C (2000). Infections urinaires nosocomiales sur sonde en réanimation: physiopathologie, épidémiologie et prophylaxie. Annales Françaises d'Anesthésie et de Réanimation..

[14] Méar JB, Kipnis E, Faure E (2013). Les interactions entre Candida albicans et Pseudomonas aeruginosa: au-delà d'une association de malfaiteurs?. Médecine et Maladies Infectieuses..

[15] Lambert PA (2002). Mechanisms of antibiotic resistance in Pseudomonas aeruginosa. Journal of the royal society of medicine..

[16] Albanèse J, Durbec O, Martin C (1996). Antibiothérapie empirique en réanimation. Conférences d'actualisation..

[17] Floret D, Dumont C (1988). Complications neurologiques des otites. Médecine et Maladies Infectieuses..

[18] Brun-Buisson C (2005). Risques et maîtrise des infections nosocomiales en réanimation: texte d'orientation SRLF/SFAR. Réanimation..

